# How dry is dead? Evaluating the impact of desiccation on the viability of the invasive species *Cissus quadrangularis*

**DOI:** 10.1002/pei3.70011

**Published:** 2024-10-15

**Authors:** Ariadna Mondragón‐Botero, Jennifer S. Powers

**Affiliations:** ^1^ Plant and Microbial Biology University of Minnesota Saint Paul Minnesota USA

**Keywords:** desiccation tolerance, ecological restoration, invasive species, Madagascar, management, tropical dry forest

## Abstract

*Cissus quadrangularis* is a succulent vine that degrades forests where it is not native by growing over trees and causing them to break or by impeding regeneration. Methods for its control have been tried but no satisfactory approach has been found yet. We carried out an experiment to analyze how much desiccation *Cissus* can endure before losing its ability to grow when rehydrated, using fragments of 0.5, 1, 2, and 3 internodes to test if desiccation tolerance was affected by fragment length. We found that Cissus remains viable after losing up to 80% of its weight, with shorter fragments losing viability (capacity to grow) at 70% weight loss. No fragments sustained viability at 90% water loss, establishing a critical threshold for *Cissus* desiccation tolerance. Our study also showed that shorter fragments (0.5 internodes) were less viable compared to longer ones (1, 2 or 3 internodes). *Cissus* has a remarkable tolerance to desiccation. Therefore, management strategies should ensure complete dehydration of *Cissus* fragments to prevent its regrowth. Reducing fragments to smaller sizes could amplify the effectiveness of control measures by reducing their viability, but risks of increasing propagule numbers should be considered.

## 1 INTRODUCTION

Biological invasions are a major threat to biodiversity worldwide, and their management usually represents a significant economic burden, with global efforts costing an average of US$26.8 billion annually (Diagne et al., 2021; IPBES, 2023). While some invasive species are deeply rooted in cultural practices and have an important economic or social value (Soubeyran, 2008), they can also have negative effects. These effects include reducing biodiversity, outcompeting native flora and fauna, altering habitat structure, disrupting ecosystem functioning and services (Diagne et al., 2021; IPBES, 2023). An example of such plant is *Cissus quadrangularis* L. (Vitaceae), a widespread succulent vine, native to the dry areas of Arabia, Africa, India, Sri Lanka, Malaysia, and Thailand. *Cissus quadrangularis* (hereafter *Cissus*) has been used traditionally to treat various diseases and it is consumed in Southern India and Sri Lanka (Bafna et al., 2021). However, *Cissus* has been introduced to Hawai'i, Mayotte, the National Park Îles de la Madeleine in Senegal, and Madagascar, where it has been listed as invasive (Nzengue et al., 2015; Soubeyran, 2008; Staples et al., 2000; Winchester et al., 2018). This plant spreads rapidly and overruns surrounding vegetation, forming dense canopies that block light and impede the growth and regeneration of native species. *Cissus* can spread both vertically and horizontally, forming dense, monospecific patches that dominate and displace local fauna and flora (Kaur & Malik, 2014; Raju & Raju, 2022). Despite various control efforts, no fully effective solutions have been developed either for its eradication or for the management of the substantial biomass it produces (Nzengue et al., 2015, 2016; Winchester et al., 2018).

*C. quadrangularis* is a succulent, shrubby climber characterized by its four‐angled stems, which are jointed at the nodes. The internodes typically measure 8 to 10 cm in length and 1.2 to 1.5 cm in width. This species propagates both sexually and asexually. Sexual reproduction involves seeds, whereas asexual reproduction primarily occurs through stem cuttings or fragments. Flowering and fruit formation are rare, making stem cuttings the predominant mode of propagation (Bafna et al., 2021; Kaur & Malik, 2014). Additionally, internodes can break apart and independently regenerate. The leaves of *Cissus* are simple, situated at the nodes with opposing tendrils that facilitate climbing onto surrounding plants. *Cissus* contributes to forest degradation by climbing over mature trees, disrupting their structure, and often breaking them, and inhibiting seedling regeneration, and it can also displace native fauna, like birds from its nesting sites (Nzengue et al., 2016; Winchester et al., 2018).

Cissus control has been attempted primarily using biological and mechanical methods (Nzengue et al., 2015, 2016). The larvae of the moth *Hippotion celerio* have been used in biological control trials, with limited success because the larvae's rate of tissue consumption is insufficient against the plant's biomass (Nzengue et al., 2015). Mechanical control methods have consisted in manually removing *Cissus* either through cutting or hand pulling followed by simply leaving the plant on site to desiccate post‐removal. This strategy, however, has proven ineffective and is potentially risky as the plant can grow readily from cuttings and reinvade the zone (Nzengue et al., 2015). The most aggressive and moderately effective approach has been manual removal followed by its incineration. However, the costs and associated risks make it a less desirable solution, leaving the need for a safer and more sustainable management strategy of the plant biomass after its removal. In this context, composting emerges as a promising sustainable approach to biomass management, mirroring successful strategies used for other succulent and/or invasive species (Alami et al., 2021; Brito et al., 2013; Liu et al., 2022; Sembera et al., 2019).

Succulent plants like *C. quadrangularis* exhibit remarkable drought tolerance which can be achieved through several mechanisms. They can alter their photosynthetic processes, conserve water, and maintain low metabolic activity during drought (Hanscom & Ting, 1978). One key response is the maintenance of cellular osmotic balance, achieved by synthesizing and accumulating compatible solutes, or “osmolytes”, in the cytoplasm to prevent cellular dehydration (Koźmińska et al., 2019). In species like *Aloe*, it has been shown that the composition of cell wall polysaccharides plays a vital role in water storage and drought response (Ahl et al., 2019). *C. quadrangularis* is known for its resistance to drought stress. *Cissus* performs CAM photosynthesis in the stems and C3‐like in the leaves, which is particularly adaptive. The leaves appear only during the rainy season, and are shed under drought conditions, leaving only the succulent stems (Raju & Raju, 2022). Moreover, a transcriptome analysis during drought conditions showed enriched pathways related to cutin, suberin, and wax metabolism in the stem, further contributing to its drought adaptation (Li et al., 2024). The capacity to tolerate drought raises legitimate concerns that inadequately composted fragments might contribute to the species' spread if they retain viability. This underscores the need for a clear understanding of the species' dehydration tolerance, specifically, whether *Cissus* can survive and potentially regrow after substantial water loss, to ensure that composting does not inadvertently facilitate further invasion.

Toward the goal of improving management options for *Cissus*, our study aims to determine whether drying and composting *Cissus* after its removal from trees is an effective management strategy and to what extent reducing the plant into smaller fragments can decrease its viability. Specifically, we address the following question:

How much dehydration can *C. quadrangularis* resist before losing its ability to grow when rehydrated, and is this tolerance a function of the fragment length? We hypothesize that *Cissus* fragments of shorter length will be less viable after desiccation, potentially serving as a control method prior to composting. We hypothesized that shorter fragments would lose their ability to grow, and thus fragmenting *Cissus* in small pieces could be a viable control method. The outcomes of this research can inform sustainable management strategies and offer alternatives to the current practices for managing this invasive vine.

## 2 METHODS

### 2.1 Desiccation experiment

We generated the biological material for the study by propagating *Cissus* from cuttings obtained from a plant in the College of Biological Sciences Plant Conservatory at the University of Minnesota. *Cissus* was grown in the greenhouse in 0.9‐gallon pots containing a mix of 40% black soil, 30% sand, and 30% composted pine bark, and fertilized once with 18 grams with Osmocote Plus, a slow‐release fertilizer (15 N:9P:11 K). The plants were well watered until the start of the experiment. To determine the tolerance to desiccation of *Cissus* and its potential to regenerate once rehydrated, we cut fragments of *Cissus* of different numbers of internodes (3, 2, 1 or half internodes) and dried them at ambient temperature (mean 21.89°C, range from 19.69°C to 26.29°C, and SE 0.01°C) (Figure S2). Each fragment was weighed at the beginning of the experiment, and reweighed weekly, until it had lost 10%, 20%, 30%, 40%, 50%, 60%, 70%, 80%, or 90% of the initial mass. We calculated this mass loss as (initial mass‐final mass)/initial mass × 100. For each mass loss category, we used 15 fragments belonging to each of one of the four fragment categories (3, 2, 1 or half internodes). After reaching a specific mass loss category, the fragments were repotted in 0.3‐gallon pots containing the soil mix described previously and were fertilized with 3 g of Osmocote. The plants were placed in a greenhouse at an average temperature of 21.89°C (range from 19.69° to 26.29°C, and SE 0.01°C) and fully watered weekly. Pots were placed in different benches in the greenhouse and rotated occasionally to avoid spatial autocorrelation biases. After at least 3 months, we harvested the plants and inspected them for the presence of shoot and/or root growth. We considered that the plant was viable if it had any signs of growth (root or shoot growth) and recorded the data as a binary response (yes = growth, no = no growth). We fitted a generalized linear model (GLM) with a binomial family across all mass loss categories and a second GLM with a binomial family to estimate the likelihood of regrowth lineal as a function of both internode number and the percentage of mass loss (Hosmer et al., 2013). The analysis was carried out in R version 4.3.1 (R Core Team, 2023).

## 3 RESULTS

**FIGURE 1:**
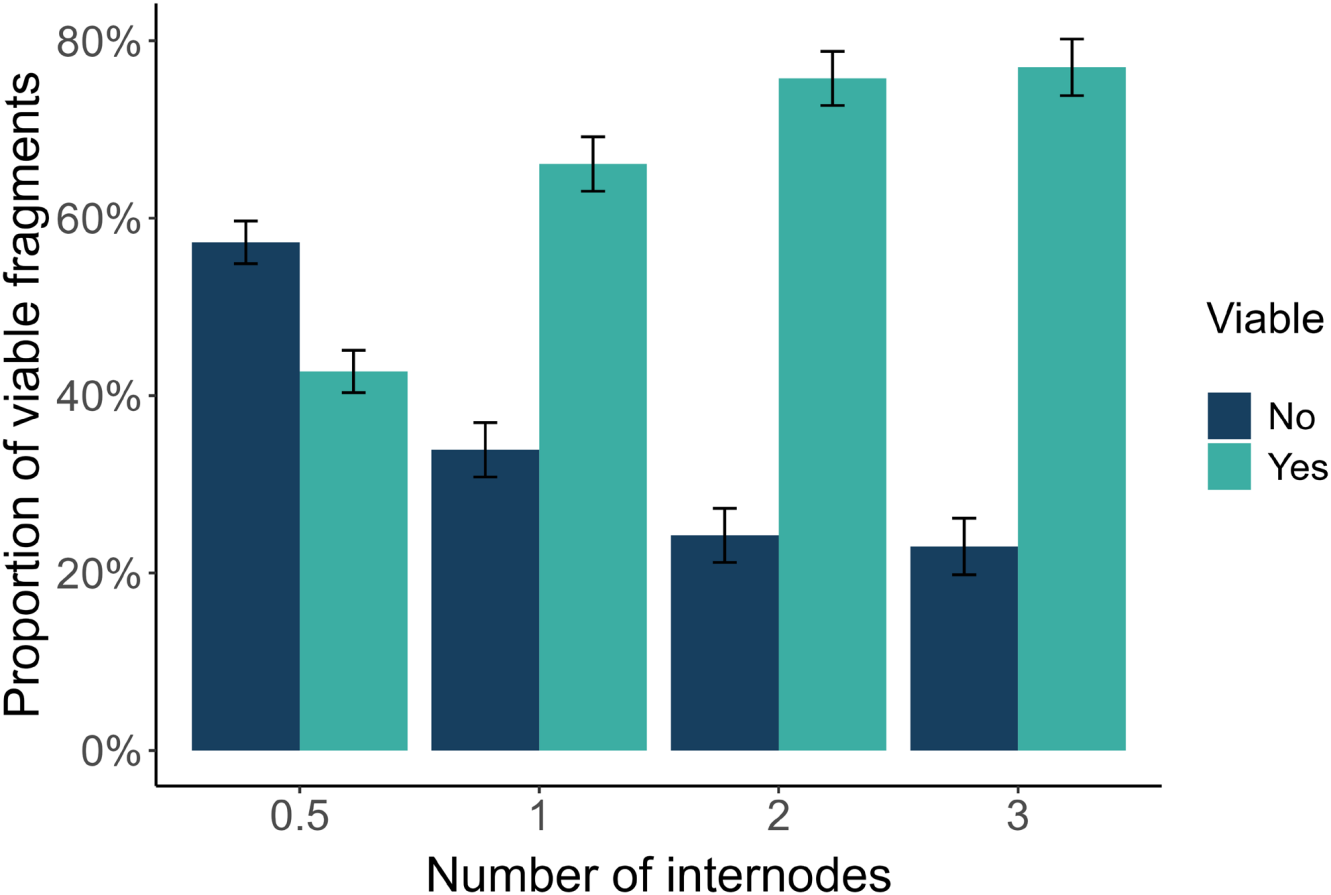
Proportion of *Cissus quadrangularis* fragments that exhibited viability (scored as regrowth of roots or shoots) for the three internode categories across all the mass loss categories (of all the 750 fragments used in the experiment). Error bars represent the standard error of the proportions. The bar plot shows the direct observations from the dataset, while the model‐based probabilities presented in the results section, derived from logistic regression, serve to estimate the likelihood of regrowth for these categories.

Our study showed that under greenhouse conditions (~22°C) *Cissus* fragments require a substantial amount of time to dry. It took up to 411 days for some fragments to lose 90% of the original mass. The logistic regression analysis revealed a significant association between the number of internodes and the probability of regrowth in *Cissus*, when compared across all mass loss categories. In other words, as the number of internodes increased, so did the viability of the *Cissus* fragments (Figure 1). Specifically, plants with 0.5 internodes had a baseline probability of regrowth of 42.7%. By comparison, plants with one internode had a 72.3% probability of regrowth, which corresponds to an increase in the log‐odds of viability of 0.961 (*z* = 5.74, *p* < .001). Plants with two internodes had an 80.7% probability of regrowth (log‐odds increase of 1.4; *z* = 7.42, *p* < .001), and it was 81.8% for plants with three internodes (log‐odds increase of 1.5; *z* = 7.31, *p* < .001).

**FIGURE 2:**
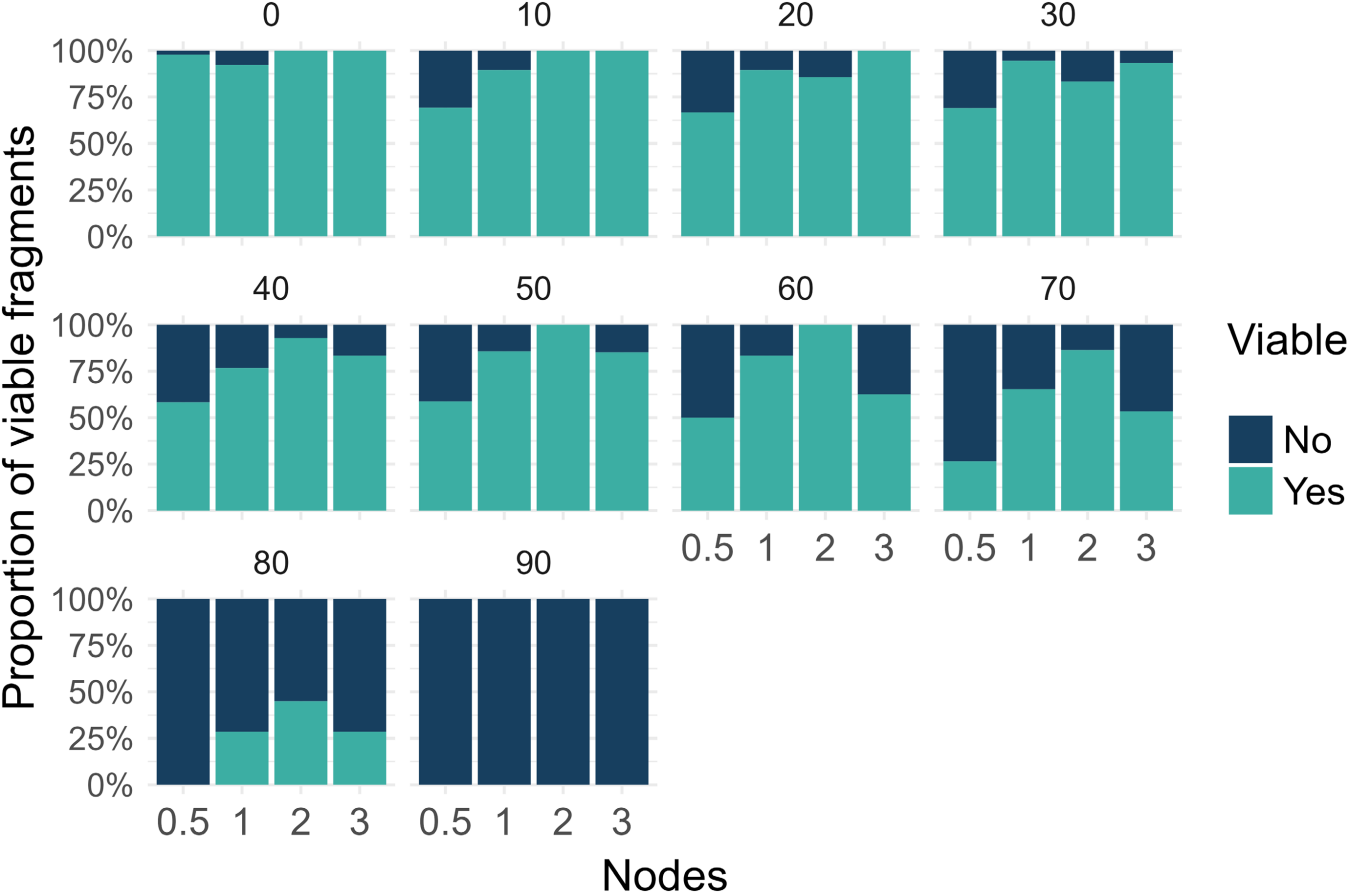
Proportion of viable (Yes) and non‐viable (No) *Cissus quadrangularis* fragments categorized by the percentage of weight loss. Numbers above the bars represent the weight loss category. Each bar represents the proportion of viable and non‐viable fragments within each length category: 0.5, 1, 2, or 3 internode‐long fragments.

The viability of *Cissus* fragments differed significantly across internode categories as weight loss increased (Figure 2, Table S1). The log‐odds of viability decreased notably beyond 70% weight loss, with the sharpest decline observed at 80% (*z* = −8.09, *p* < .001) across all internode categories. Fragments with 0.5 internodes had a marked decrease in viability at 70% weight loss, and fragments with one, two, and three internodes maintained viability even at 80% water loss, indicating a substantial resilience that may have implications for the species' invasiveness. For example, *Cissus* could remain viable under extreme environmental stress, potentially facilitating its spread and establishment in new areas. No fragments were viable following 90% water loss, indicating a threshold beyond which regrowth is highly unlikely.

Fragments with two internodes exhibited greater viability than those with three internodes, notably at desiccation levels ranging from 40% to 80%. This finding was unexpected, as it could be assumed that a greater number of internodes would correlate with increased water reserves and, consequently, enhanced survival. Hence, we investigated potential interaction effects between the number of internodes and various levels of desiccation. However, the statistical analysis revealed no significant interaction effects for fragments with either two or three internodes across the range of mass loss categories examined (Table S2).

## 4 DISCUSSION AND CONCLUSIONS

Our study showed that *Cissus* has a remarkable ability to remain viable after significant water loss and can regrow even from small fragments. This ability complicates the management strategies in regions where it has become invasive. Our most striking result was that *Cissus* can retain viability after losing 80% of its mass. As previous studies have found that the water content of *Cissus* at full hydration is 93% (Venkatasami et al., 2023), most of the observed mass loss corresponds to water loss. This high‐water content likely facilitates its survival up to a critical threshold. However, beyond 90% mass loss, viability sharply declines, indicating that there is a limit to its desiccation tolerance. Previous research had also shown that *Cissus* is very tolerant to drought. This tolerance could be linked to an enhanced expression of genes related to cutin, suberin, and wax metabolism during drought conditions (Li et al., 2024). Future research on the mechanisms of drought resistance could be key to design better targeted strategies for the management of this species.

Internode length impacted *Cissus* viability as well. Although cutting the plant in smaller pieces reduces its viability, half internode fragments can remain viable even after a 70% weight loss. Reducing fragment size is a moderately effective but labor‐intensive measure for accelerating desiccation and controlling this species once removed from the invaded areas. However, there is an inherent risk: smaller fragments could increase the number of potential propagules, enhancing the plant's ability to spread. Thus, while effective to a degree, reducing fragment size introduces a complex balance between reducing viability and inadvertently increasing propagation potential. Thus, this risk needs to be considered when managing *Cissus* biomass in the invaded areas. Although current literature does not report desiccation‐induced dormancy in *Cissus*, the potential for dormancy to impact our measures of viability remains a consideration and merits further exploration (Costa et al., 2016). Finally, more internodes might be expected to provide greater water reserves, thus enhancing survival under water stress. However, our findings do not support this assumption. More internodes might generally mean more resources for survival, but also higher metabolic costs or different water‐use efficiencies that affect desiccation tolerance. Further physiological studies could elucidate these mechanisms, possibly exploring aspects like cuticular resistance or photosynthetic activity during stress.

Succulent species management carries its difficulties due to the ability to retain water in the stem and be viable for long periods. For example, a study showed that after 6 months of storage, plant fragments of the invasive succulent *Carpobrotus edulis* were able to survive, rehydrate, grow and produce new roots, indicating that viability and consequently, the capacity of *C. edulis* to colonize new habitats or reappear in restored habitats remained intact (Souza‐Alonso et al., 2019). Several plants belonging to the plant family Cactaceae (commonly referred to as cacti) are widespread invasive alien species in different parts of the world. These invasive cacti, like *Cissus*, have succulent stems and the ability to propagate asexually. For these cacti, physical control methods were the methods most frequently reported as being used. Such methods involve the physical removal of plants, followed by treatment and burial. Options for treating the removed plants before burying them include placing them in water for a minimum of 16–20 days (to promote rotting) or drying or burning them at a minimum of 2 meters above the surface, to avoid reshooting. However treated areas need to be monitored for several years to detect regrowth and achieve complete eradication (Novoa et al., 2019).

In situations where it is feasible, burning *Cissus* biomass is more effective than biological control to manage the large quantities of biomass produced by the plant (Nzengue et al., 2015). In theory, composting would be a better alternative to burning because the end product could be used to enrich the soil in organic matter (Sembera et al., 2019). In light of our results, composting could be used after *Cissus* is removed from the invaded areas, provided that the conditions ensure that the plant material is non‐viable. According to our results, this means ensuring that all the fragments have lost at least 90% of their weight, reaching almost complete desiccation. Given the period it takes *Cissus* to desiccate at 50°C (a temperature attained during composting), composting over several months could guarantee that the plant material is not viable, particularly if coupled with methods that break the plant into smaller fragments. If composting is to be done, we recommend it is done in an enclosed facility, or placing a fence around an open‐air composting area to minimize the risk of *Cissus* fragments spreading.

Our results do suggest some recommendations. The methods used to eradicate invasive species vary with available resources and site constraints (Holl, 2020). The most cost‐effective strategy is prevention, but early detection, eradication and control are all part of their management strategies, as have been shown for the management of other succulent species (IPBES, 2023; Novoa et al., 2019; Souza‐Alonso et al., 2019). Additionally, it is important to monitor the invaded areas regularly after initial removal efforts to detect and manage any regrowth.

Management strategies for *Cissus* should prioritize the complete removal of the plant from the invaded area, ensuring that the area is completely free from fragments, given the species' ability to propagate from small pieces, such as even half internodes. Our study demonstrated that even these small fragments can retain significant viability and contribute to re‐invasion if not thoroughly eradicated, which underscores the need for meticulous removal practices for *Cissus*. After removal, complete desiccation needs to be ensured, considering its ability to retain viability after substantial water loss. This could be achieved by containing and covering the plant material with tarps to amplify the sun's heat. However, we recommend further research to validate the efficacy of this strategy. Given that *Cissus* colonizes degraded and open areas (Winchester et al., 2018), habitat restoration following *Cissus* removal is recommended whenever possible, to minimize recolonization of the species. While the controlled greenhouse conditions in our study led to valuable insights, field studies are essential to test the effectiveness of desiccation as a management strategy in natural settings. With the growing commercial interest in *Cissus*, cultivation of this species could increase (Bafna et al., 2021). Therefore, we recommend caution to prevent potential invasions in other ecosystems.

## FUNDING INFORMATION

This project was carried out with funding from the U.S. Department of Energy, Office of Science, Office Biological and Environmental Research, under Award Number DESC0020344.

## CONFLICT OF INTEREST STATEMENT

The authors have no conflict of interest to declare.

## ONE SENTENCE SUMMARY

*Cissus quadrangularis* viability decreased markedly after 80% of mass loss, especially for shorter fragments.

## Supporting information


Figure S1.

Figure S2.

Table S1.

Table S2.

## Data Availability

The data that support the findings of this study are openly available in doi:10.5061/dryad.gtht76hvn at https://datadryad.org/.
