# Supplementary figures and images for: How dry is dead? Evaluating the impact of desiccation on the viability of the invasive species Cissus quadrangularis
Source: Plant Environ Interact. 2024 Oct 15;5(5):e70011. doi: 10.1002/pei3.70011 (PMC11474622; doi:10.1002/pei3.70011)

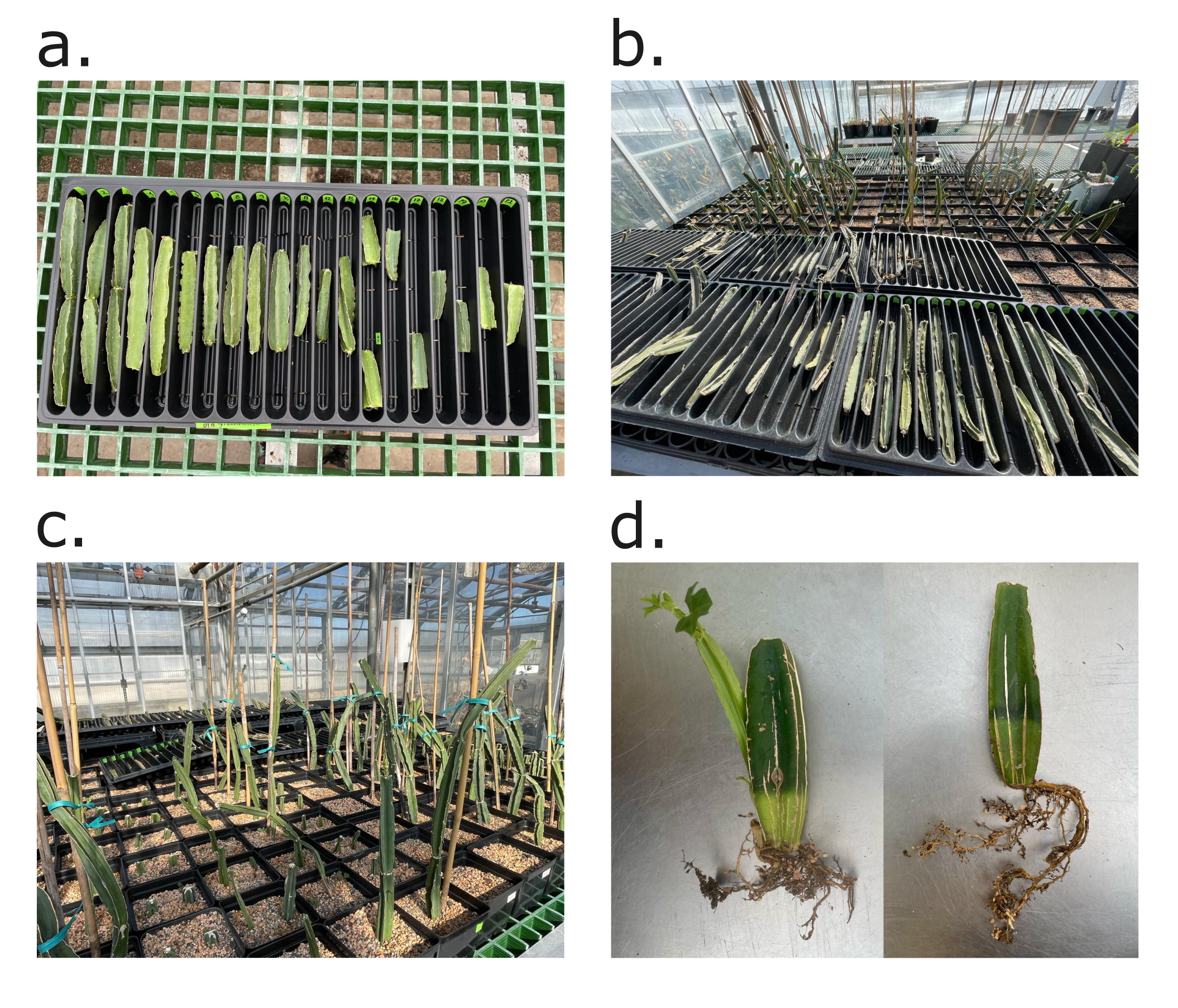

Supplement: Supplementary file 1 — Figure S1. [file PEI3-5-e70011-s004.tif]

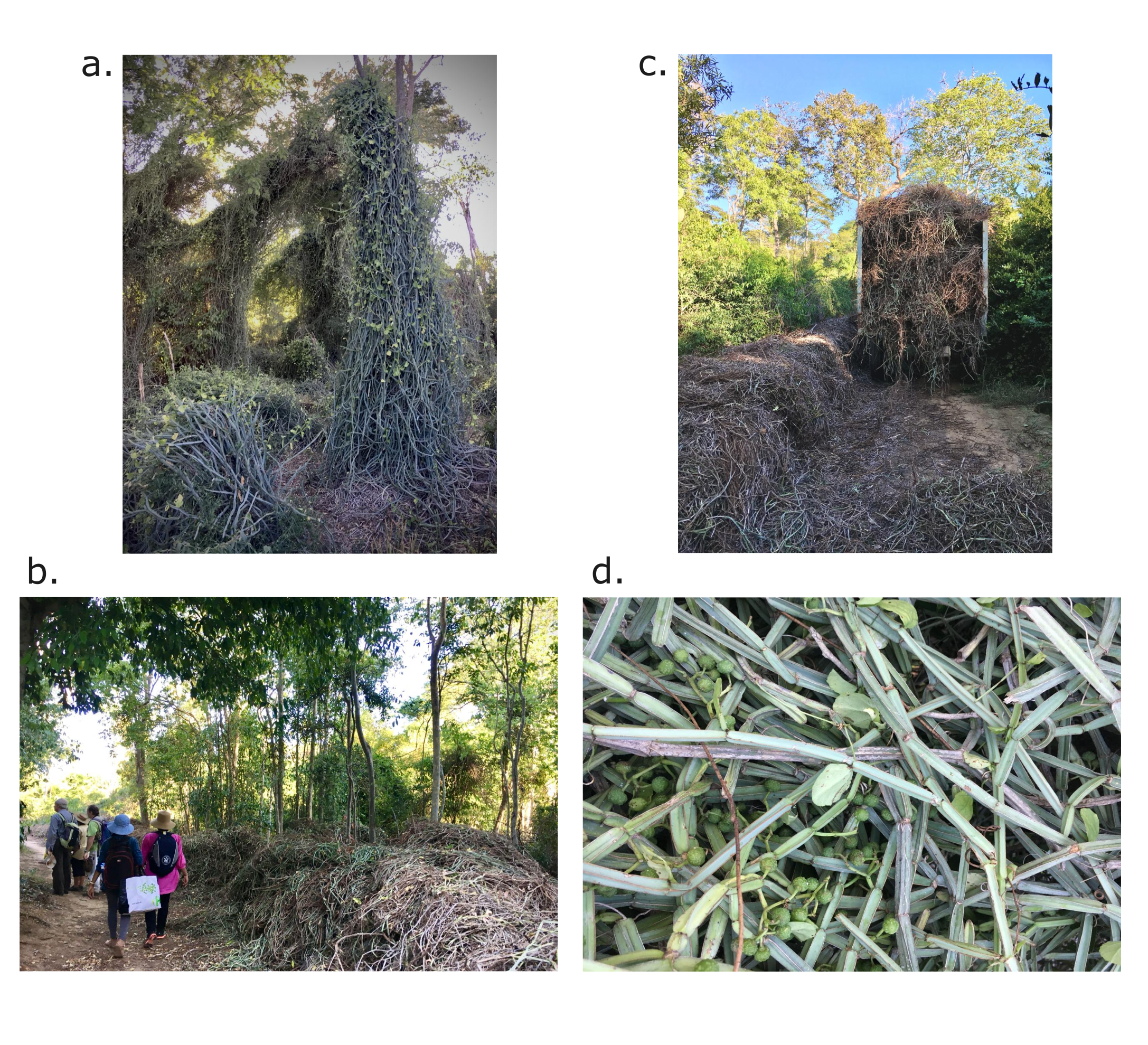

Supplement: Supplementary file 2 — Figure S2. [file PEI3-5-e70011-s002.tif]
