# Supplementary material for: How dry is dead? Evaluating the impact of desiccation on the viability of the invasive species Cissus quadrangularis
Source: Plant Environ Interact. 2024 Oct 15;5(5):e70011. doi: 10.1002/pei3.70011 (PMC11474622; doi:10.1002/pei3.70011)
Supplement: Supplementary file 3 — Table S1. [file PEI3-5-e70011-s003.docx]

| **Category** | **Estimate** | **Std. Error** | **z value** | **Pr(>\|z\|)** |
| --- | --- | --- | --- | --- |
| 0% mass loss | 2.97 | 0.59 | 5.03 | < 0.001 |
| 10% mass loss | -1.87 | 0.68 | -2.73 | 0.006 |
| 20% mass loss | -2.33 | 0.65 | -3.58 | < 0.001 |
| 30% mass loss | -2.23 | 0.65 | -3.43 | < 0.001 |
| 40% mass loss | -2.79 | 0.63 | -4.38 | < 0.001 |
| 50% mass loss | -2.54 | 0.64 | -3.96 | < 0.001 |
| 60% mass loss | -3.08 | 0.64 | -4.82 | < 0.001 |
| 70% mass loss | -3.85 | 0.63 | -6.07 | < 0.001 |
| 80% mass loss | -5.55 | 0.68 | -8.08 | < 0.001 |
| 90% mass loss | -22.50 | 436.50 | -0.05 | 0.96 |
| 1 node | 1.36 | 0.24 | 5.56 | < 0.001 |
| 2 nodes | 2.32 | 0.32 | 7.22 | < 0.001 |
| 3 nodes | 1.35 | 0.27 | 5.08 | < 0.001 |
