# Supplementary material for: How dry is dead? Evaluating the impact of desiccation on the viability of the invasive species Cissus quadrangularis
Source: Plant Environ Interact. 2024 Oct 15;5(5):e70011. doi: 10.1002/pei3.70011 (PMC11474622; doi:10.1002/pei3.70011)
Supplement: Supplementary file 4 — Table S2. [file PEI3-5-e70011-s001.docx]

| **Category** | **Estimate** | **Std. Error** | **z value** | **Pr(>\|z\|)** |
| --- | --- | --- | --- | --- |
| 0% mass loss | 3.807 | 1.011 | 3.765 | 0.000 |
| 10% mass loss | -2.996 | 1.097 | -2.732 | 0.006 |
| 20% mass loss | -3.114 | 1.071 | -2.907 | 0.004 |
| 30% mass loss | -3.004 | 1.065 | -2.822 | 0.005 |
| 40% mass loss | -3.470 | 1.066 | -3.255 | 0.001 |
| 50% mass loss | -3.450 | 1.069 | -3.226 | 0.001 |
| 60% mass loss | -3.807 | 1.071 | -3.554 | 0.000 |
| 70% mass loss | -4.828 | 1.083 | -4.457 | 0.000 |
| 80% mass loss | -23.373 | 2292.763 | -0.010 | 0.992 |
| 90% mass loss | -23.373 | 989.988 | -0.024 | 0.981 |
| 1 internode | -1.322 | 1.251 | -1.057 | 0.291 |
| 2 internodes | 15.759 | 2404.671 | 0.007 | 0.995 |
| 3 internodes | 15.759 | 2150.803 | 0.007 | 0.994 |
| 10% mass loss: 1 internode | 2.651 | 1.518 | 1.747 | 0.081 |
| 20% mass loss: 1 internode | 2.769 | 1.499 | 1.847 | 0.065 |
| 30% mass loss: 1 internode | 3.353 | 1.654 | 2.028 | 0.043 |
| 40% mass loss: 1 internode | 2.175 | 1.365 | 1.593 | 0.111 |
| 50% mass loss: 1 internode | 2.757 | 1.406 | 1.961 | 0.050 |
| 60% mass loss: 1 internode | 2.931 | 1.445 | 2.028 | 0.043 |
| 70% mass loss: 1 internode | 2.979 | 1.373 | 2.170 | 0.030 |
| 80% mass loss: 1 internode | 19.972 | 2292.764 | 0.009 | 0.993 |
| 90% mass loss: 1 internode | 1.322 | 1949.559 | 0.001 | 0.999 |
| 10% mass loss: 2 internodes | 2.996 | 3322.530 | 0.001 | 0.999 |
| 20% mass loss: 2 internodes | -14.661 | 2404.671 | -0.006 | 0.995 |
| 30% mass loss: 2 internodes | -14.952 | 2404.671 | -0.006 | 0.995 |
| 40% mass loss: 2 internodes | -13.531 | 2404.671 | -0.006 | 0.996 |
| 50% mass loss: 2 internodes | 3.450 | 4036.826 | 0.001 | 0.999 |
| 60% mass loss: 2 internodes | 3.807 | 3547.578 | 0.001 | 0.999 |
| 70% mass loss: 2 internodes | -12.892 | 2404.671 | -0.005 | 0.996 |
| 80% mass loss: 2 internodes | 3.606 | 3322.530 | 0.001 | 0.999 |
| 90% mass loss: 2 internodes | -15.759 | 3348.215 | -0.005 | 0.996 |
| 10% mass loss: 3 internodes | 2.996 | 3442.964 | 0.001 | 0.999 |
| 20% mass loss: 3 internodes | 3.114 | 3442.964 | 0.001 | 0.999 |
| 30% mass loss: 3 internodes | -13.923 | 2150.803 | -0.006 | 0.995 |
| 40% mass loss: 3 internodes | -14.486 | 2150.803 | -0.007 | 0.995 |
| 50% mass loss: 3 internodes | -14.367 | 2150.803 | -0.007 | 0.995 |
| 60% mass loss: 3 internodes | -15.249 | 2150.803 | -0.007 | 0.994 |
| 70% mass loss: 3 internodes | -14.604 | 2150.803 | -0.007 | 0.995 |
| 80% mass loss: 3 internodes | 2.890 | 3143.679 | 0.001 | 0.999 |
| 90% mass loss: 3 internodes | -15.759 | 4014.916 | -0.004 | 0.997 |
